# Bioinformatics screening of biomarkers related to liver cancer

**DOI:** 10.1186/s12859-021-04411-1

**Published:** 2021-10-25

**Authors:** Ye-Cheng Wang, Zhen-Bo Tian, Xu-Qing Tang

**Affiliations:** 1 School of Science, Jiangnan University, Wuxi, 214122 China; 2 Wuxi Engineering Research Center for Biocomputing, Wuxi, 214122 China

**Keywords:** Bioinformatics analysis, Differential expressed genes, PPI, Oncomine, Survival curve analysis

## Abstract

**Background:**

Liver cancer is a common malignant tumor in China, with high mortality. Its occurrence and development were thoroughly studied by high-throughput expression microarray, which produced abundant data on gene expression, mRNA quantification and the clinical data of liver cancer. However, the hub genes, which can be served as biomarkers for diagnosis and treatment of early liver cancer, are not well screened.

**Results:**

Here we present a new method for getting 6 key genes, aiming to diagnose and treat the early liver cancer. We firstly analyzed the different expression microarrays based on TCGA database, and a total of 1564 differentially expressed genes were obtained, of which 1400 were up-regulated and 164 were down-regulated. Furthermore, these differentially expressed genes were studied by using GO and KEGG enrichment analysis, a PPI network was constructed based on the STRING database, and 15 hub genes were obtained. Finally, 15 hub genes were verified by applying the survival analysis method on Oncomine database, and 6 key genes were ultimately identified, including PLK1, CDC20, CCNB2, BUB1, MAD2L1 and CCNA2. The robustness analysis of four independent data sets verifies the accuracy of the key gene’s classification of the data set.

**Conclusions:**

Although there are complicated differences between cancer and normal cells in gene functions, cancer cells could be differentiated in case that a group of special genes expresses abnormally. Here we presented a new method to identify the 6 key genes for diagnosis and treatment of early liver cancer, and these key genes can help us understand the pathogenesis of liver cancer more deeply.

## Background

As one of the common malignant tumors in China, liver cancer accounts for 70–80% of primary liver malignancies, and are the second leading cause of cancer-related mortality [1–3]. Current treatments for liver cancer mainly include surgery and intervention, etc. However, as the underlying mechanism of liver cancer is still unclear, liver cancer has a high incidence, strong invasiveness and poor prognosis. Data show that about 70% of liver cancers relapse within 5 years after resection or ablation [4]. Early detection and treatment can effectively improve the prognosis of tumor patients and reduce the mortality rate, but there are usually no obvious clinical symptoms in the early stage of liver cancer. At present, the commonly used diagnostic methods of liver cancer, such as serological tumor markers and imaging techniques, are not satisfactory in clinical effect. Therefore, screening markers related to early diagnosis, invasion and metastasis of liver cancer is of great significance for improving the therapeutic effect and prognosis of liver cancer [5].

In this study, the original data was downloaded from the TCGA database, and the R software was used to screen the differentially expressed genes (DEGs) in the gene expression profile of liver cancer tissues and adjacent tissues. Using bioinformatics technology to analyze DEGs, combined with Oncomine database to screen potential molecular targets suitable for early diagnosis and immunotherapy of liver cancer [6]. Survival analysis and literature mining were used to verify our results and provided a theoretical basis for further research. Finally, we constructed a clinical prediction model to get more convincing results.

## Results

According to the mRNAs matrix data of normal tissues and liver cancer tissues in the TCGA database, using the R toolkit, letting $\alpha =2$ and $\beta =0.05$, a total of 1564 DEGs were obtained, of which 1400 were up-regulated and 164 were down-regulated.

1564 DEGs were enriched by GO and KEGG with DAVID, where $\beta =0.01$ was included as inclusion criteria in GO, and *p* value $<0.05$ was included as inclusion criteria in KEGG. The results of GO enrichment analysis indicated that the DEGs were mainly enriched in BPs, including sister chromatid cohesion and chemical synaptic transmission. CC showed that the DEGs were significantly enriched in extracellular region, chromosome, extracellular space, plasma membrane and anchored component of membrane, and centromere region. MF showed that the DEGs were significantly enriched in hormone activity, sequence-specific DNA binding, growth factor activity and calcium ion binding. The results of KEGG pathway enrichment analysis showed that the DEGs were mainly enriched in Cell cycle, Neuroactive ligand-receptor interaction, Hypertrophic cardiomyopathy, Mineral absorption, Dilated cardiomyopathy, Adrenergic signaling in cardiomyocytes and Cardiac muscle contraction (Seen in Table 1).

**Table 1:** Significantly enriched GO terms and KEGG pathways of DEGs

Category	Term	Description	Count	FDR/p value
BP term	GO:0007268	Chemical synaptic transmission	44	$2.26e-04$
BP term	GO:0007062	Sister chromatid cohesion	24	$4.38e-03$
CC term	GO:0005576	Extracellular region	198	$2.66e-08$
CC term	GO:0005615	Extracellular space	158	$1.08e-04$
CC term	GO:0031225	Anchored component of membrane	26	$1.86e-03$
CC term	GO:0005886	Plasma membrane	386	$1.16e-02$
CC term	GO:0000775	Chromosome, centromeric region	16	$2.77e-02$
MF term	GO:0043565	Sequence-specific DNA binding	79	$2.65e-06$
MF term	GO:0005179	Hormone activity	24	$4.46e-04$
MF term	GO:0005509	Calcium ion binding	88	$6.24e-03$
MF term	GO:0008083	Growth factor activity	29	$3.83e-02$
KEGG pathway	hsa04080	Neuroactive ligand–receptor interaction	46	$2.33e-07$
KEGG pathway	hsa04110	Cell cycle	23	$8.06e-05$
KEGG pathway	hsa04978	Mineral absorption	12	$2.43e-04$
KEGG pathway	hsa05410	Hypertrophic cardiomyopathy (HCM)	16	$4.47e-04$
KEGG pathway	hsa04260	Cardiac muscle contraction	15	$9.36e-04$
KEGG pathway	hsa04261	Adrenergic signaling in cardiomyocytes	22	$1.00e-03$
KEGG pathway	hsa05414	Dilated cardiomyopathy	16	$1.01e-03$

The STRING database was used to construct a protein-regulated interaction network diagram and 1498 protein interaction network nodes were obtained. Analyze these nodes to further understand the regulatory relationship between DEGs and proteins in liver cancer tissues and normal tissues. Import the data downloaded in the STRING database into Cytoscape software for visualization, and get the PPI network diagram of DEGs. The degree of CytoHubba plug-in was used to determine the top 15 hub genes for further analysis. They were GNGT1, GNG4, LPAR3, BDKRB1, PLK1, CDC20, CCNB2, ADCY8, BUB1, MAD2L1, AURKB, AGTR2, RLN3, CCNA2, and CENPE.

**Fig. 1:**
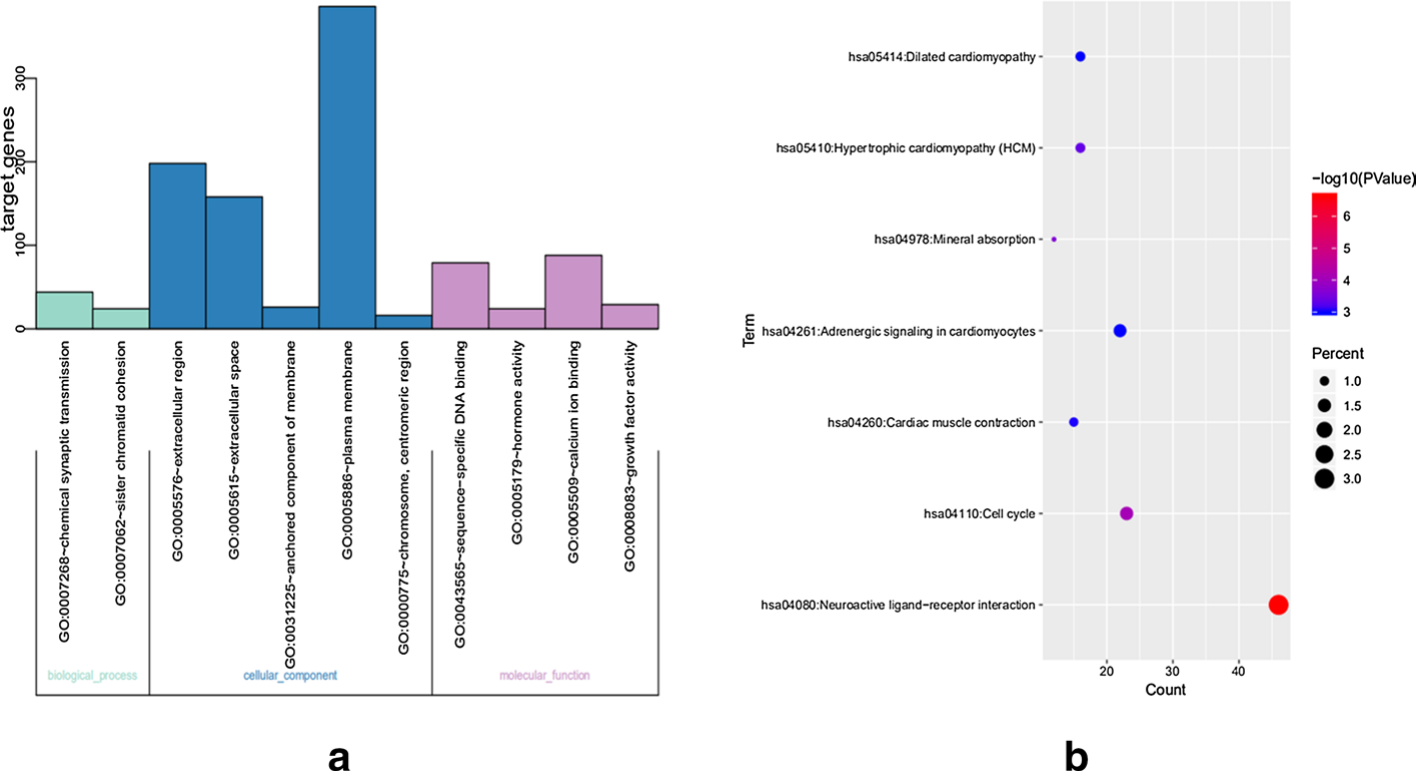
Differential expression analysis of CDC20 gene in Oncomine database liver cancer. Where: 1. normal tissue; 2. liver cancer tissue; a, b, c respectively represents the results of three studies

**Fig. 2:**
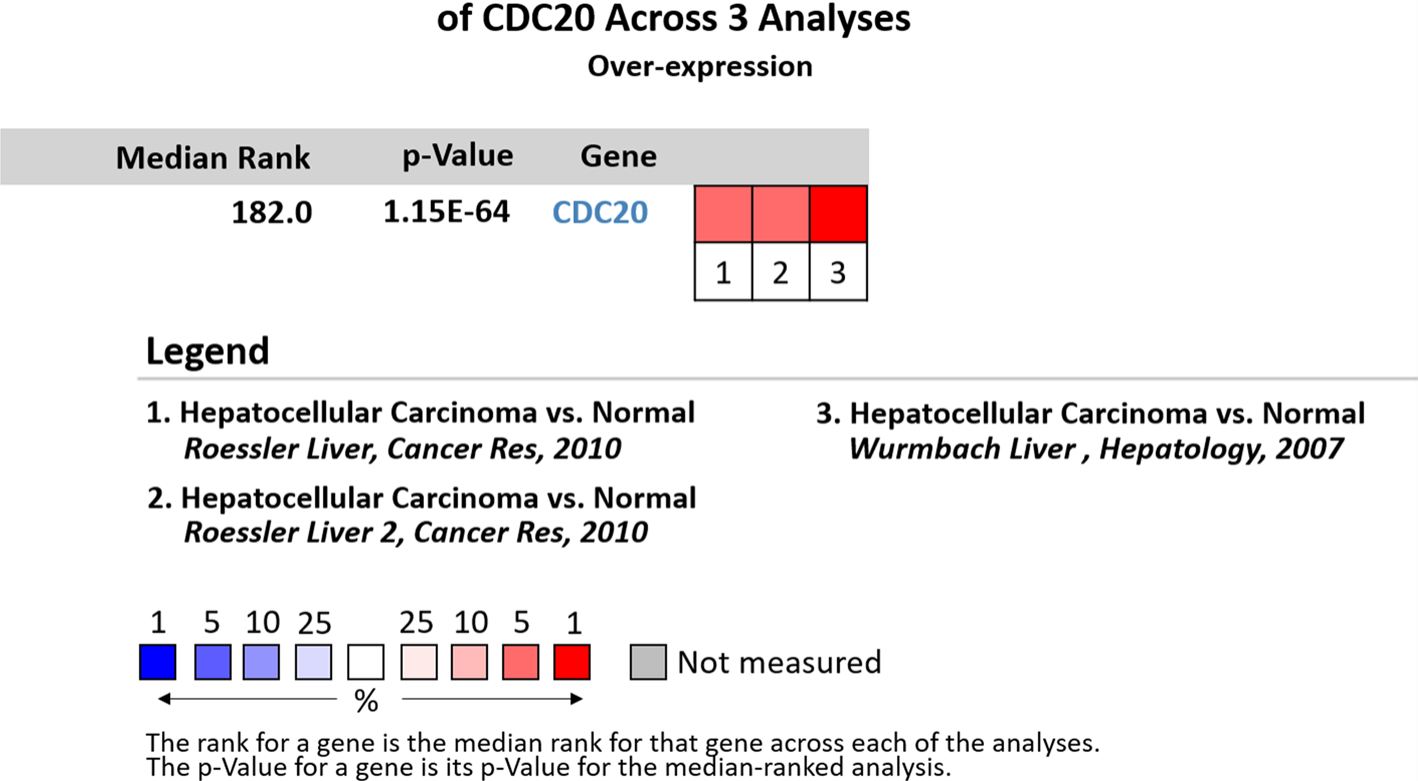
Meta-analysis of Oncomine database of CDC20 gene in liver cancer

The expression levels of these 15 hub genes were analyzed in the Oncomine database, and we obtained 6 key genes, PLK1, CDC20, CCNB2, BUB1, MAD2L1 and CCNA2, which have significant expression differences, as shown in Fig. 1. Furthermore, a meta-analysis of these 6 key genes was carried out. Taking CDC20 gene as an example, the results of differential expression analysis and meta-analysis are shown in Fig. 2. In the meta-analysis, three studies were screened by significant differences and differential expression folds [7, 8]. Depending on the statistical analysis of Oncomine database, 6 key genes were highly expressed in liver cancer compared with normal tissues, and the differences were statistically significant. Next, we used the 5-fold cross-validation method and three classifiers: decision tree(Dtree), random forest(RF), and support vector machine(SVM) to measure the classification performance of 6 key genes and 15 hub genes in 4 data sets. Taking into account the randomness of the classifier, the process was repeated 200 times and the average accuracy was calculated. The results are shown in Table 2.

**Fig. 3:**
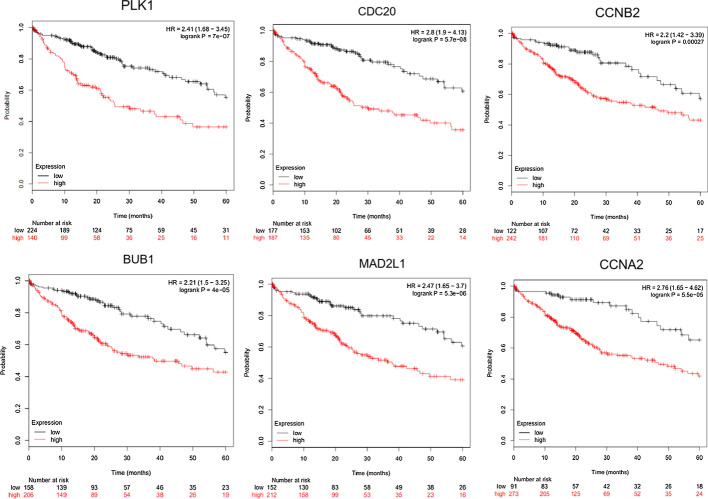
The results of KM-Plotter database survival analysis on 6 key genes

Finally, using the KM-Plotter database, the survival curves were obtained based on the 6 key genes. Conducting research on the prognostic value of these key genes for the overall survival of patients with liver cancer, the results shown that: PLK1 ($P=7e-07$), CDC20 ($P=5.7e-08$), CCNB2 ($P=2.7e-04$), BUB1 ($P=4e-05$), MAD2L1 ($P=5.3e-06$) and CCNA2 ($P=5.3e-06$). The analysis results of survival curve are shown in Fig. 3. These results show that these six key genes are closely related to the occurrence, development and prognosis of liver cancer, which were significant for the study of liver cancer.

**Fig. 4:**
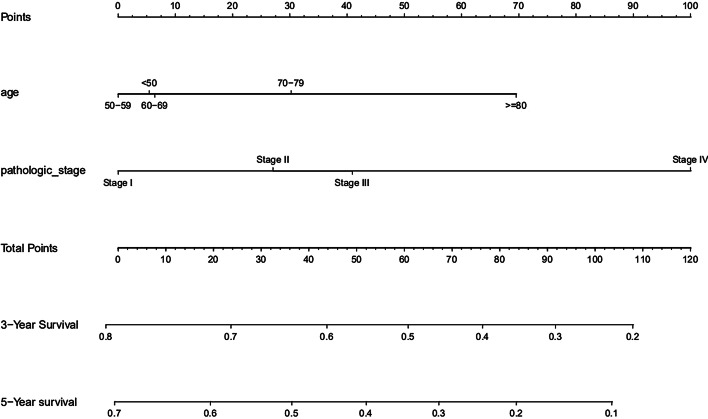
Liver cancer overall survival prediction nomogram

**Fig. 5:**
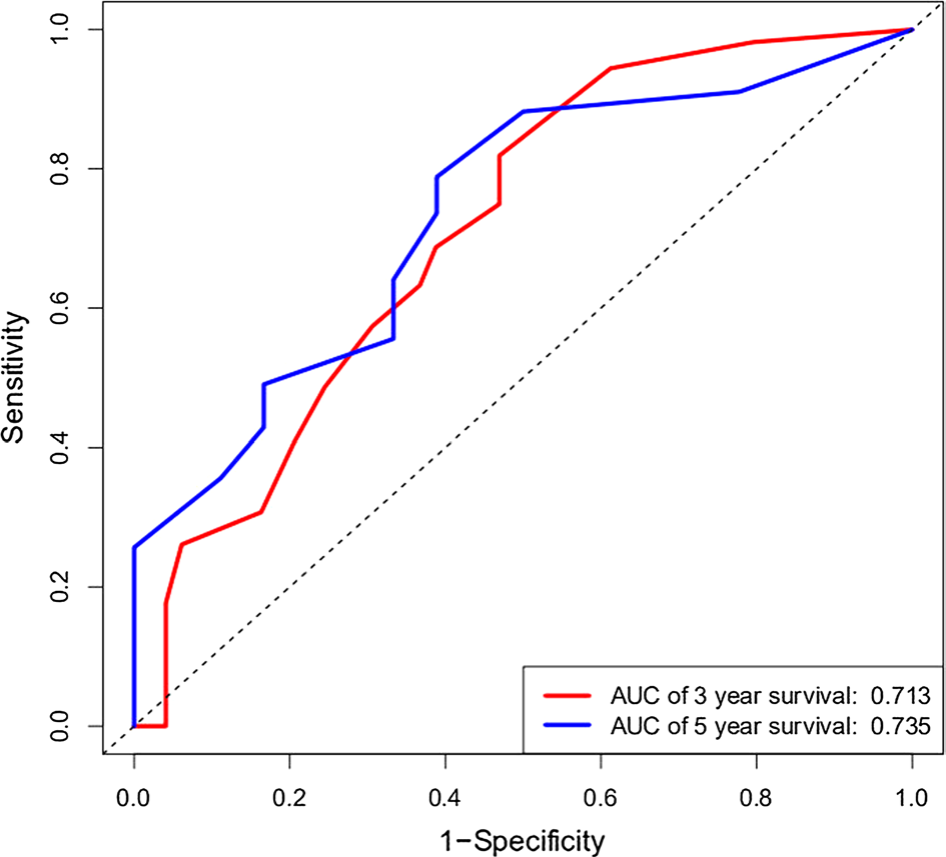
ROC of the risk score

Based on the clinical prediction model, a univariate cox regression analysis was performed on the training group data set, and the p value of age and pathology was less than 0.05. Multivariate cox regression analysis was performed for these two factors, and a nomogram was drawn as shown in Fig. 4. The ROC curve of the training group (seen in Fig. 5) and the validation results of the test population show that the model has good prediction ability.

## Discussions

It is a dynamic biological process with multiple molecules,steps and factors during the occurrence and development of liver cancer, and people’s understanding of its mechanism is still very limited presently. Previous studies mainly focused on the impact of single genes on tumors, but the process of cell carcinogenesis often involves changes in multiple genes. These genes can play a role by transforming network interactions. Therefore, studying cancer gene expression profile at the level of multiple genes can help us better explore the pathogenesis. Gene chip can detect the expression levels of tens of thousands of genes at the same time, and is a powerful tool for studying the interaction between genomes and genes.

In this study, to verify the performance of the 6 key genes in classifying cancer and normal samples, four datasets from different sources were applied. By using different classifiers for verification, higher classification accuracy has been obtained. By comparing the classification accuracy of 15 hub gene sets and 6 key gene sets, we can find that the prediction accuracy of the two gene sets is not much different, but because the number of genes was reduced, the speed of clinical diagnosis can be improved. Therefore, our method has strong robustness and effectiveness. Compared with normal samples all of the 6 key genes are upregulated. Of the 6 key genes, PLK1 plays an important role in the regulation of cell mitosis and is closely related to survival and prognosis. In recent years, it has been found that PLK1 is highly expressed in cervical cancer [9], neuroblastoma cells [10], acute myeloid leukemia [11], prostate cancer [12] and many other malignant tumors. Studies have found that inhibiting the expression of PLK1 by interfering with multiple stages of mitosis can lead to tumor cell death, so PLK1 is expected to become a potential target for cancer treatment [13].

CDC20 is a key gene to ensure the normal progress of cell mitosis. Its abnormal expression can cause normal cells to produce non-integer chromosomes by disrupting cell mitosis, thereby promoting malignant transformation of cells [14]. Studies have shown that the high expression of CDC20 is closely related to the clinical progress of human malignant tumors [15, 16], such as lung cancer, liver cancer, malignant glioma, etc [17–19].

The proteins encoded by CCNA2 and CCNB2 belong to the highly conserved cyclin family and play an important role in the process of cell cycle control. The research results of the literature [20, 21] show that they are all highly expressed in HCC tissues, and the overall survival rate of patients is low.

BUB1 encodes a serine/threonine-protein kinase that play an important role in mitosis [22]. The protein partly encoded by this gene regulates the formation and activation of the late mitotic complex by phosphorylating some components of the mitotic checkpoint complex and activating the checkpoint of the spindle [23, 24]. The abnormal expression of BUB1 is related to the occurrence and development of a variety of tumors, and it is also involved in the biological behavior of cancer stem cells such as breast cancer [25, 26].

MAD2L1 is an important part of the spindle assembly checkpoint in mitosis. Studies have shown that reducing the expression of MAD2L1 can inhibit tumor cell migration and invasion, thereby hindering tumor occurrence and development [27].

In the process of cell carcinogenesis, the interaction of multiple gene changes is of great significance to the pathogenesis of cancer, not only for liver cancer. Therefore, the analysis method in this study is also applicable to other cancers and is used to identify biomarkers of other complex human diseases, such as the non-coding RNA, microRNAs, etc [28–30].

## Conclusions

This study comprehensively explored the molecular functions and biological processes of the occurrence and development of liver cancer. Based on the analysis of the liver cancer data from the TCGA database, firstly compared the gene expression of normal tissues and liver cancer tissues, and screened out genes with different expressions. The GO enrichment analysis and KEGG pathway enrichment analysis of these differential genes shown that DEGs were mainly enriched in chemical synaptic transmission, extracellular area, extracellular space, neuroactive ligand-receptor Interaction, cell cycle, etc. Then, the PPI network of DEGs was constructed using STRING database and Cytoscape software, and 15 hub genes were screened out. In addition, in order to verify the expression levels of these key genes, differential expression analysis and meta-analysis of 15 hub genes were performed using Oncomine database, and 6 key genes were obtained, and the fivefold verification method was used to verify the effectiveness of these six key genes. Finally, Oncomine and survival analysis were used to confirm the six key genes that PLK1, CDC20, CCNB2, BUU1, MAD2L1 and CCNA2 are closely related to the occurrence, prognosis and mechanism of liver cancer, which will help us discover tumor markers and drug targets.

## Methods

### Datasets

The mRNA data of liver cancer tissues and normal tissues were downloaded from the TCGA database. The data includes 371 tumor samples and 50 normal samples. The four gene expression profiles (GSE76427 [31], GSE57957 [32], GSE39791 [33], GSE102079 [34]) used for the verification test were obtained from the Gene Expression Comprehensive System (GEO) [35] Download.

### Methods

This paper aims to find molecular markers for early diagnosis of liver cancer and potential molecular targets for liver cancer immunotherapy. The methods used in the study were as follows: Screening for the DEGs, GO and KEGG enrichment analysis, PPI network construction and hub genes identification, Oncomine validation and survival analysis of hub genes. These methods were described in detail as follows:

#### Screening for the DEGs

Using the R package, we performed background correction, standardization and expression value calculation on the original data. Index $\mid logFC \mid \>\alpha$ and $FDR < \beta$ were used as conditions to filter out DEGs, where the value of $\alpha$ was inversely proportional to the number of DEGs selected, and the value of $\beta$ was proportional to the number of DEGs selected.

#### GO and KEGG enrichment analysis

GO is a method for large-scale enrichment studies of gene functions including biological processes, molecular functions, and cellular components. Statistical analysis was used to calculate the p value and FDR in GO. KEGG pathway enrichment analysis calculates the hypergeometric distribution of DEGs and pathways, and returns the p value and FDR value of each pathway existing in DEGs to find the most likely related pathway. In this study, we used DAVID 6.8 database for GO and KEGG enrichment analysis. Then visually analyze GO and KEGG through bar graphs and ggplot2.

#### PPI network construction and hub genes identification

The STRING database is an online analysis tool used to study protein-protein interaction (PPI) patterns and provide information on related pathways and functions [36]. Import DEGs into the database and output network files of protein interactions. Then use the Cytoscape software to visualize the protein interaction network and obtain the hub genes [37, 38].

#### Oncomine validation and survival analysis of hub genes

The Oncomine database is a cancer gene chip database used to analyze cancer gene information, compare the differential expression between cancer and normal tissues, and verify hub genes. The KM-plotter database (http://kmplot.com/analysis/) contains information on 364 liver cancer patients and is used to evaluate the prognostic value of hub genes in liver cancer patients.

#### In silico validation

In order to verify the validity of the results, we downloaded 4 independent gene expression profiles of GEO (GSE76427, GSE57957, GSE39791, GSE102079). The five-fold cross-classification verification method was adopted, and the four data sets were respectively verified using decision trees, random forests and support vector machines. Each experiment was repeated 200 times and the average classification accuracy was calculated.

#### Construction of clinical prediction model

The clinical data of liver cancer samples were randomly divided into training group and testing group. Univariate cox regression analysis was performed using the training group to screen out statistically significant factors and draw the histograms. According to the calculation results, the training group was divided into high-risk and low-risk categories, and the ROC curve was constructed to evaluate the model. The testing group was used to verify the model.

In this study, the DEGs were obtained by differential expression analysis of liver cancer gene expression profiles. These differential expression genes were studied by GO and KEGG enrichment analysis, and the significantly enriched functions and pathways of DEGs were obtained. Furthermore, protein interaction network was constructed using STRING database to screen key genes. Oncomine database and survival analysis were used to confirm the key genes, and the genes which suitable for liver cancer markers or potential targets were obtained. Finally, we constructed a clinical prediction model, so as to get more convincing results.

**Table 2:** The classification accuracy of 15 hub genes and 6 key genes using 3 classifiers in 4 data sets

Dataset	Number of genes	Classifier and classification accuracy		
		Dtree	RF	SVM
GSE76427	15hub	0.850	0.919	0.832
	6key	0.878	0.907	0.827
GSE57957	15hub	0.894	0.917	0.861
	6key	0.893	0.908	0.856
GSE39791	15hub	0.916	0.911	0.887
	6key	0.925	0.915	0.915
GSE102079	15hub	0.854	0.888	0.898
	6key	0.843	0.859	0.875

## Data Availability

Not applicable.

## Abbreviations

DEG: Differentially expressed genes
GO: Gene ontology
KEGG: Kyoto encyclopedia of genes and genomes
DAVID: The database for annotation, visualization and integrated discovery
PPI: Protein–protein interaction
FC: Fold change
FDR: False discovery rate
KM-Plotter: Kaplan–Meier Plotter database
STRING: STRING database
